# Effort-Related Decision-Making in ADHD

**DOI:** 10.20900/jpbs.20200027

**Published:** 2020-12-25

**Authors:** Suzanne H. Mitchell, Deborah Sevigny-Resetco

**Affiliations:** 1 Department of Behavioral Neuroscience, Oregon Health & Science University, Portland, OR 97239-3098, USA; 2 Department of Psychiatry, Oregon Health & Science University, Portland, OR 97239-3098, USA; 3 Oregon Institute of Occupational Health Sciences, Oregon Health & Science University, Portland, OR 97239-3098, USA

**Keywords:** ADHD, effort discounting, cognitive effort, decision-making, motivation, executive function, attention, working memory

## Abstract

ADHD is defined by behavioral symptoms that are not well characterized in relation to ADHD’s neurobiological mechanisms. This approach has limited our ability to define ADHD nosology and predict outcomes because it does not systematically examine facets of the disorder such as the inability to maintain cognitively effortful activities, as promoted in the NIMH RDoC approach. Existing data indicate ADHD is associated with differences in reward valuation and processing, but we do not know whether ADHD is also associated with higher levels of aversion to exerting cognitive effort and/or altered reward x effort interactions. Our ongoing study addresses this knowledge gap by examining individuals’ preferences between rewards associated with minimal effort and reward alternatives with a higher payoff but higher effort costs (“effort discounting”); thereby permitting us to characterize differences in biases and tradeoffs during effort-related decision-making in ADHD. The study takes advantage of a well-defined sample of ADHD-diagnosed and healthy control individuals to address three aims. First, we determine whether ADHD is associated with steeper discounting of larger, more effortful rewards. Second, we examine the subjective perception of effort in youth diagnosed with ADHD and healthy controls using tasks requiring varying levels of cognitive effort. Third, we explore relationships amongst indices of effort discounting, theoretically-related traits (e.g., grit, distress tolerance), biomarkers of effort-related decision-making (eye movements and pupil size), and various cognitive measures. Successful completion of the aims will permit us to better characterize ADHD-healthy control differences and lay a foundation for more computational approaches to ADHD diagnostic criteria.

## INTRODUCTION

The inability or reluctance to engage in cognitive (mental) effort is a cross-cutting feature of numerous psychopathologies and related to the Research Domain Criteria (RDoC) construct: Reward Valuation; subconstruct: Effort [1]. One such psychopathology, Attention Deficit Hyperactivity Disorder (ADHD), has been strongly associated with this RDoC construct, and it is a cardinal concern raised by parents, teachers, and ADHD-diagnosed individuals themselves. However, full characterization of the factors and mechanisms explaining the inability or unwillingness to remain engaged in specific activities such as schoolwork or homework is lacking, retarding our understanding of this disorder and the development of targeted interventions.

In the last few years, interest in the neurobiological mechanisms underlying effort-related decision-making has been growing, but most of these studies have examined physical effort, rather than cognitive effort, e.g., squeezing handgrips [2–5], typing letter strings [6], finger tapping [7] and have focused on healthy control samples or rodent models. A single study, Mies et al. [8], compared decision-making preferences with physical effort in ADHD and healthy control samples, but reported no group differences. It is our contention that, although interesting information can be gained from studying physical effort, its correlation with decision-making for cognitive effort is limited [9] and studies focused on cognitive effort are more likely to yield data that will enhance our conceptualization of the behavioral symptoms of ADHD.

In terms of component cognitive mechanisms, previous research in ADHD has focused on reward valuation and reward processing [10,11], on control of sustained attention [12,13] and on executive function [14,15]. However, these approaches neglect to operationalize the critical component of cognitive effort, a central process model of both cognitive control [16] and reward valuation [17]. The present research will address this knowledge gap by adapting procedures successfully used to assess the role of delay on cognitive control of impatience and reward valuation: “delayed reward discounting” or more commonly “delay discounting” or “intertemporal choice”. Delay discounting procedures assess biases and tradeoffs when choosing between small, immediate rewards versus larger, delayed rewards [18], and meta-analyses indicate that ADHD is associated with heightened delay discounting [19,20]. Thus, it seems reasonable from a theoretical standpoint to use a similar procedure to examine individuals’ preferences between rewards requiring minimal cognitive effort and rewards with higher payoffs but higher effort costs (“effort discounting”). The literature on “effort discounting” is significantly less developed than that for delay discounting and so there are no well-established procedures to manipulate cognitive effort used in this context. However, from a practical standpoint, it seems reasonable to expect that cognitive effort can be varied by manipulating requirements to engage and maintain executive functions, whether that be attention, updating/working memory, set-shifting or inhibitory processes [21].

A few studies exist that support this expectation, although none examined individuals diagnosed with ADHD. Botvinick et al. [7] required subjects to choose between performing blocks of trials in which there was either a low- or high-cognitive-effort requirement, and effort was varied by requiring more frequent task-shifting between performing a magnitude judgment task and performing an ink-color identification (Stroop) task. Westbrook et al. [22] developed a task in which individuals performed an *N*-back memory task, then made decisions between small and large rewards associated with a low or high *N*-back requirement. Data from these studies, and research using hypothetical tasks [23], indicate that the subjective value of the larger, more effort-requiring reward decreases systematically as the cognitive effort level increases. Unfortunately, while these data support the idea that cognitive effort discounting can, in principle, be assessed in ADHD-diagnosed samples, the tasks themselves have disadvantages that make them less-than-ideal for measuring differences between potentially cognitively divergent samples: (1) there are practical limits to the number of levels of cognitive effort possible, which could make it difficult to equate subjective ratings of the cognitive effort demanded; (2) participants’ concern that they cannot perform the task accurately might drive choices, rather than the feeling that the task was do-able but that the participant did not want to put in the effort. These concerns are bolstered by a recent study by Hsu et al. [24] that examined the subjective discomfort reported by undergraduate students during the Paced Auditory Serial Addition Test [25]. Hsu et al. stated that participants who scored above threshold on the ADHD self-report scale [26] reported more discomfort and more mental effort was required to perform the task and their performance was lower than that of individuals who scored below threshold. Further, in a follow-up study using only healthy controls, these researchers reported that ratings of discomfort in the same task were correlated but distinguishable from ratings of effort exerted [27]. Our proposed work will extend the approach used in those studies to different cognitive effort tasks and assess additional, subjective dimensions to shed light on the critical aspects of cognitive load driving cognitive effort discounting. We plan to use two types of cognitive effort in order to extend our understanding of the cognitive effort construct and to enhance the rigor of the research: sustained attention and working memory engagement.

### Summary of Research Aims

#### Aim 1:

Determine whether ADHD is associated with heightened discounting of larger, more cognitively effortful rewards than found in healthy controls for tasks in which effort levels have been equated by matching subjective effort ratings.

#### Aim 2:

Examine the subjective perception of cognitive effort in youth diagnosed with ADHD and healthy controls using two tasks in which they are required to exert varying levels of cognitive effort over time (sustained attention, working memory).

#### Aim 3:

Explore relationships amongst indices of cognitive effort discounting, theoretically related traits such as grit [28] and distress tolerance [29], biomarkers of effort-related decision-making (eye movements and pupil size) and measures of cognitive function assessed during a separate visit as part of another study of the same participants.

## MATERIALS AND METHODS

### Recruitment and Eligibility

All subjects are recruited from an ongoing research study at Oregon Health & Science University (ADHD heterogeneity, mechanisms, and risk profile; NIH R37 MH059105; PI: J. T. Nigg), which includes a healthy control cohort and an ADHD cohort. The healthy control cohort were identified in the Nigg research program as “typically developing” in that they had normal range IQ and no major psychiatric or medical problems, although they were allowed to have mild psychiatric disorders including anxiety disorders and dysthymia, to avoid a “super healthy” healthy control group. The ADHD cohort in the Nigg research program were identified as meeting diagnostic criteria for ADHD at baseline. Individuals represent the full range of race, ethnicity, and socio-economic characteristics of the local community in proportions similar to the local population.

With the assistance of Dr. Nigg’s study team, our study will identify and recruit 96 healthy controls and 96 ADHD-diagnosed youth from these cohorts for a single study visit using the criteria provided in Table 1.

### Study Design

**Figure 1.**
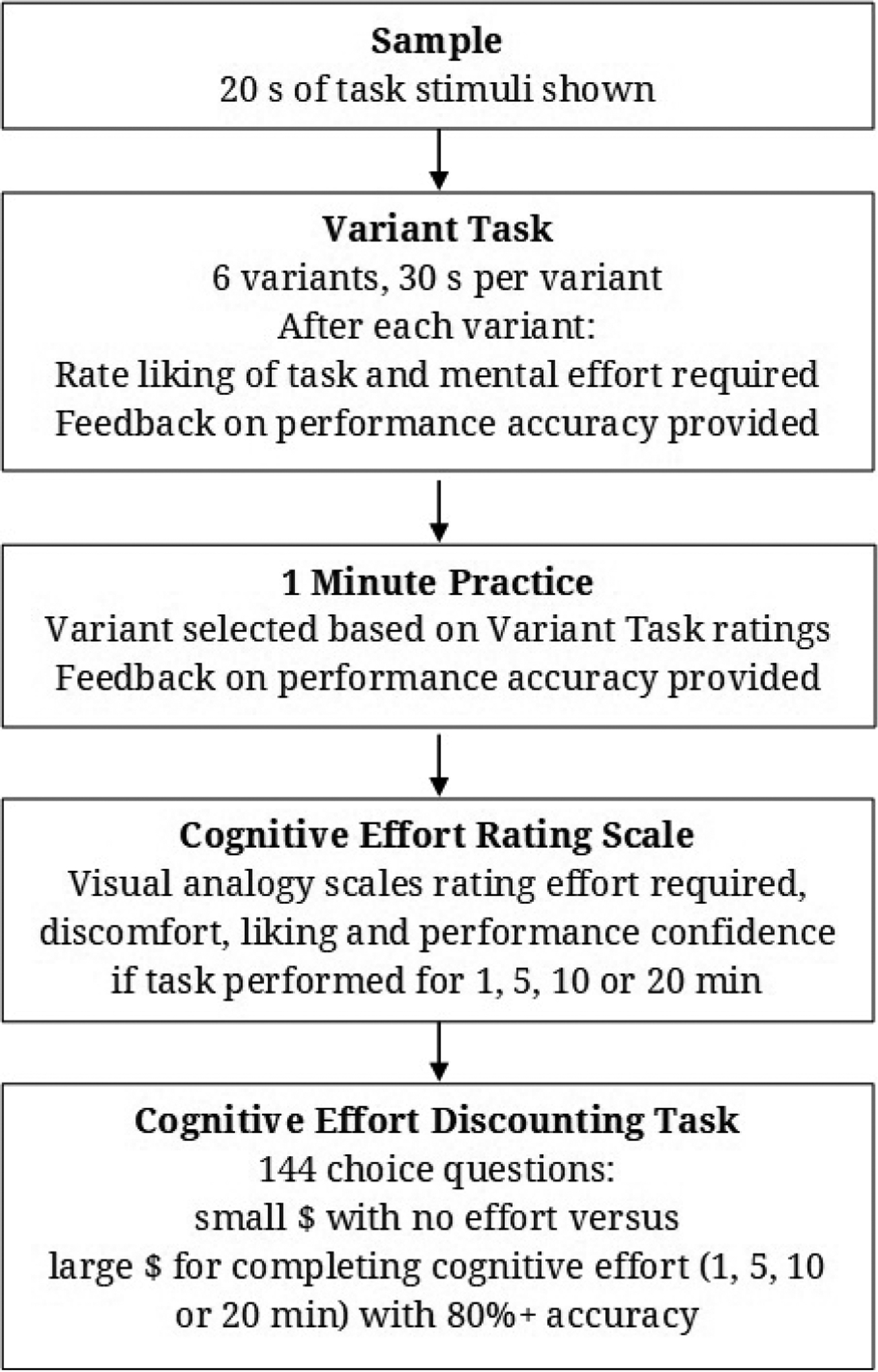
Sequence of steps to examine each type of cognitive effort.

The main focus of the project is to compare preferences between rewards requiring minimal cognitive effort and rewards with higher payoffs but higher effort costs in ADHD-diagnosed and healthy control individuals using the Cognitive Effort Discounting Task (Aim 1). To enhance study rigor, participants will perform a cognitive effort task for 1 minute prior to completing the discounting task (1 Minute Practice: Figure 1), rather than merely imagine what it would be like to complete the effort. Also, we plan to examine two types of cognitive effort using two computer tasks: one requiring sustained attention and one requiring working memory to be continuously engaged. To control for the effects of fatigue, we will counterbalance the order in which the two types of cognitive effort are examined between participants. To equate subjective levels of effort between the ADHD-diagnosed and healthy control individuals in the Cognitive Effort Discounting Task, participants will perform six different variants of each cognitive effort task for 30-s each and rate the difficulty and the subjective effort required to complete each of them (Variant Task: Figure 1). This will allow us to compare subjective ratings between ADHD-diagnosed and healthy control individuals for different stimulus presentation rates and durations (Aim 2). Block randomization will be used to vary parameter order between individuals so that all durations of stimulus presentation are examined for each inter-stimulus interval (ISI) before changing the interval. Initial ISI value will be counterbalanced between individuals. It is hoped that this will reduce variability in difficulty and effort ratings induced by successive contrast effects. To accomplish Aim 3, participants will complete a number of questionnaire measures. Further, eye tracking and pupil size will be monitored during the six different task variants in the Variant Task, and additional measures of cognitive and executive function will be drawn from assessments completed on a separate session as part of the Nigg research program.

### Study Visit Overview

At the beginning of the study visit, every participant will complete a series of questionnaires delivered using Qualtrics Survey Software (Qualtrics.com): (1) the Global Unique Identifier questionnaire required as part of the NIMH data sharing plan, (2) a demographics questionnaire, (3) a health questionnaire including information about drug use history because this is known to affect delay discounting and so may also influence cognitive effort discounting [31,32], (4) an income questionnaire [33] to provide possible covariates for the Cognitive Effort Discounting Task which uses monetary rewards, and (5) the Morning-Eveningness questionnaire [34] to provide information about participant fatigue and the participant’s circadian cycle relative to the study visit time. Detailed descriptions of all measures are in the “Assessments” section. After completing these five assessments, the participant will begin the sequence of activities associated with examining one type of cognitive effort (sustained attention or working memory).

**Figure 2.**
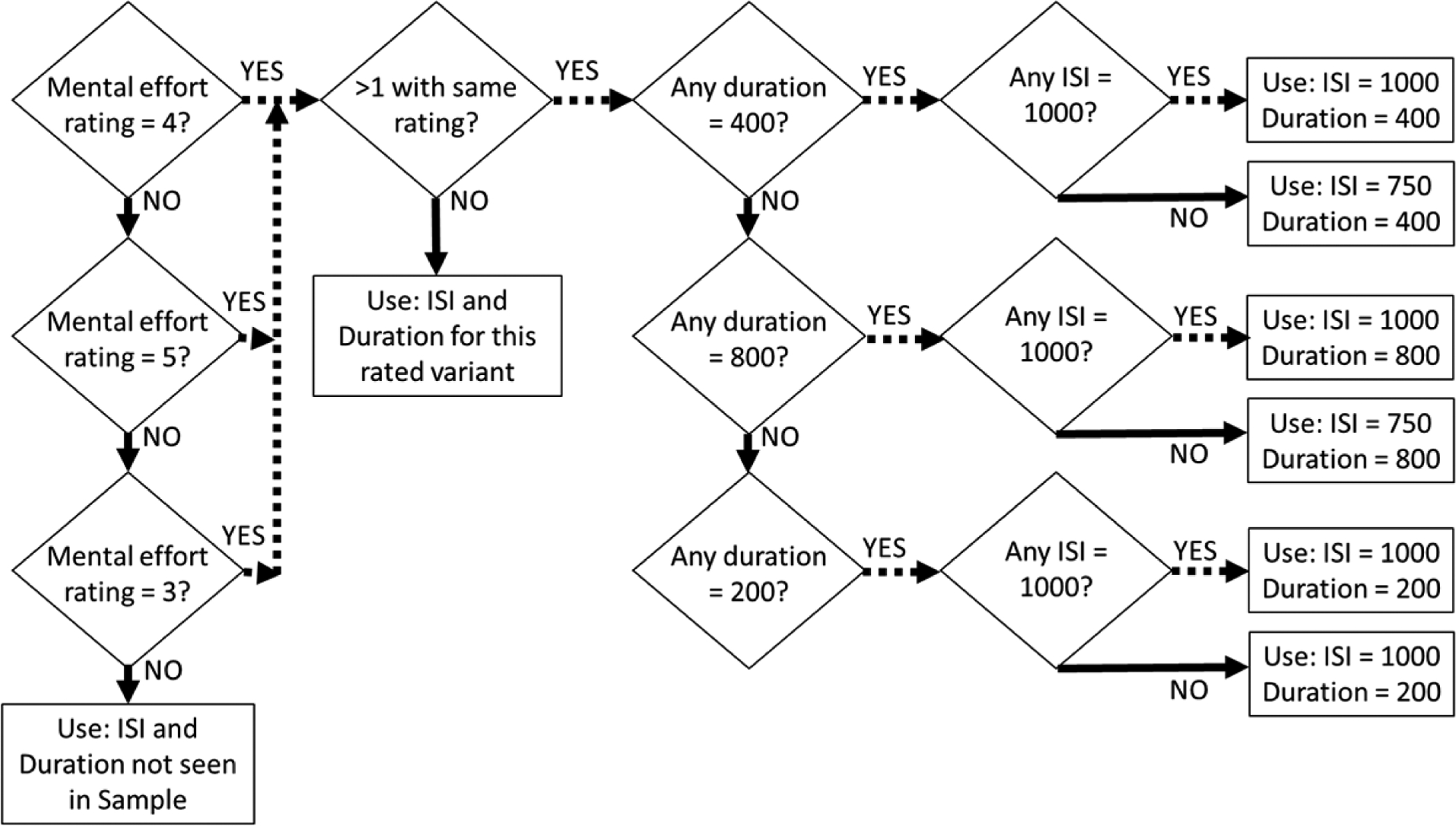
The algorithm to convert an individual’s rating for “How much mental effort was required?” in the Variant Task into the ISI and Stimulus Duration values for use in the 1 minute practice and the Cognitive Effort Discounting Task. All ISI and Stimulus Durations are in ms.

As shown in Figure 1, each sequence begins with the participant viewing a 20-s non-interactive sample of the cognitive effort task (Sample: Figure 1) so that they can become familiar with the stimuli and their presentation characteristics: ISI of either 750 or 1000 ms (matching the ISI selected to be used first in the Variant Task) and a Stimulus Duration of 400 ms. Then the participant will complete the Variant Task. This task requires the participant to perform cognitive effort for 30 s under six sets of stimulus presentation characteristics: ISI (750 or 1000 ms) and Stimulus Duration (200, 400 or 800 ms). Following each 30-s variant, the participant will answer two questions using a 7-category Likert scale: “How much did you like doing the task?” and “How much mental effort was required?” The participant will then be provided with feedback about the performance accuracy for this variant. This feedback is shown after the Likert scales are complete, so that the performance feedback does not influence responses. Responses on the Likert scales will be used to identify the ISI and Stimulus Duration parameters associated with “moderate” mental effort requirements using an algorithm (Figure 2). Then the participant be instructed that they will perform the cognitive effort task for 1 minute using those stimulus presentation characteristics that they rated as “moderate effort” to re-acquaint themselves with the level of effort it requires (1 Minute Practice). Afterwards, the participant will complete the Cognitive Effort Rating Scale, which asks the participant to imagine that they had done that task for several different time periods (1, 5, 10 or 20 min), their rating of the effort required, discomfort, liking and confidence they could perform the task with an accuracy of 80% or higher. Immediately following this, participants will complete a 144-question Cognitive Effort Discounting Task.

To create a break between completing this sequence of tasks for one type of cognitive effort and the next sequence examining the other type of cognitive effort, the participant will complete questionnaires assessing trait measures of willingness to exert and maintain effort. Then, after completing the sequence of activities associated with assessing the other type of cognitive effort (Figure 1), one of the two Cognitive Effort Discounting Tasks will be selected at random. One question from the 144 choices in that task will be randomly selected, and whichever alternative the participant chose on that question will be awarded (the small, no-effort alternative or the larger but effort-requiring alternative); although receiving the larger reward will be contingent on the participant successfully completing the specified cognitive effort requirement. An interval will be imposed before the participant is debriefed and receives the study payment so that selecting the no-effort alternative will not enable them to leave the study earlier than anyone selecting the largest cognitive effort requirement.

#### IRB approval

Initial ethical approval for this study (STUDY00019831) was obtained by the Institutional Review Boards of Oregon Health & Science University on 5/9/2019.

### Assessments

#### Questionnaire measures of participant characteristics

##### GUID Questionnaire:

Includes participant’s first/middle/last name, sex, date of birth and city of birth as noted on their birth certificate. Questionnaire is included to ensure anonymous identifier can be created for each subject, allowing submission of data to the National Institute of Mental Health Data Archive.

##### Demographics Questionnaire:

Includes 6 items to gather basic participant information on age, gender, race and ethnicity, educational level and socioeconomic status (single item based on the MacArthur Scale of Subjective Social Status [35]).

##### Morning-Eveningness Questionnaire [34]:

Includes 19 items to gauge participant preferences for activity at various times of day, e.g., “What time would you get up if you were entirely free to plan your day?” Questionnaire also provides insight into participant’s perceived levels of tiredness, alertness and willingness to engage in various actives throughout the day, e.g., “During the first half hour after you wake up in the morning, how tired do you feel?”

##### Income Questionnaire (based on Brandstätter & Brandstätter [33]):

Includes 12 items to assess the participant’s current income level, personal beliefs regarding money including degree of agreement with statements such as “Money should be spent, not saved” and hypothetical questions to assess the subjective value of some of the monetary rewards offered in the effort discounting task, e.g., “Imagine you have won $50 in the Lotto. How intense would be your joy?” using a 9-item Likert scale.

##### Health Questionnaire:

Includes 28 items to obtain a summary of the participant’s lifetime and recent (30-day) recreational drug, medication use and overall patterns of well-being, e.g., sleep quality and exercise habits.

#### Questionnaire measures of willingness to exert effort

##### Distress Tolerance Scale [29]:

Includes 14 statements related to the ability to tolerate emotional distress, e.g., “I’ll do anything to avoid feeling distressed or upset” rated on a 5-item Likert scale.

##### Persistence, Perseveration and Perfectionism Questionnaire [36]:

Includes 22 statements related to self-perceptions about task completion, e.g., “Once I have decided to do something, I keep going until I reach my goal” rated using 5-item Likert scale.

##### 12-Item Grit Scale [28]:

Includes 12 items examining perseverance of effort and consistency in maintaining goals, e.g., “I am a hard worker” rated using a 5-item Likert scale.

##### Brief Need for Cognition [37,38]:

Includes 18 statements relating to preference for engaging in cognitive activities, e.g., “Thinking is not my idea of fun” rated using 9-item Likert scale.

#### Nigg research study: cognitive measures

##### Stop Signal Task [39,40]:

Computerized task in which either an X or O stimuli appears on the screen, which the participant responds to with a key press. Throughout the task, a tone occurs intermittently indicating the participant must refrain from making the key press.

##### Spatial Span Task [41]:

Computerized task in which circle stimuli are shown one at a time within a grid in various locations. Once all stimuli have been presented, the participant is required to click on the grid boxes in either the order the circles were presented (spatial span forward) or the reverse order in which the circles were presented (spatial span backward).

##### Continuous Performance Task [42,43]:

Computerized task in which 4-digit numbers are shown sequentially on the screen. The participant is required to respond when the current number matches the previous one.

##### Reproduction of Interval by Finger Tapping [44–46]:

Computerized task in which a sequence of several tones are played for the participant with varying intervals between each tone. The participant is required to tap a button in pace with the tones while they are played and then continue to tap in the same sequence after the tones end.

##### Wechsler Intelligence Scale for Children—Fourth Edition [47]:

A standardized, written task used to identify intellectual ability level and intelligence quotients for children.

#### Cognitive effort tasks

##### Sustained Attention Task:

**Figure 3.**
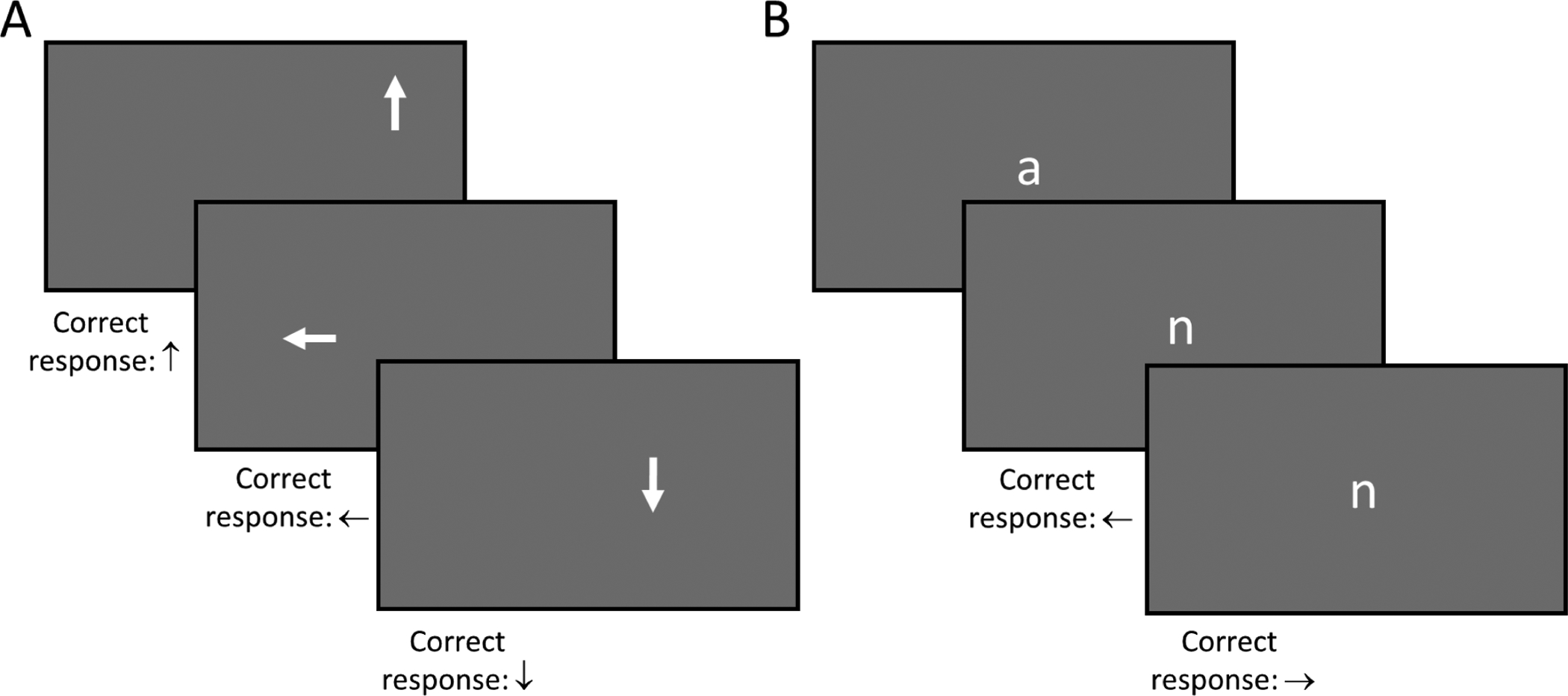
Stimulus presentations on the participant’s computer monitor (stimuli enlarged relative to monitor size for illustration) and correct responses for the Sustained Attention Task (**A**) and for the Working Memory Task (**B**).

White arrows are displayed on a grey background on the computer screen in various locations (←↑→↓; Figure 3A). Participants are asked to match each arrow stimulus presented on the screen with a corresponding arrow key press.

##### Working Memory Task:

White lower-case letters are displayed on a grey background at the center of the computer screen (Figure 3B). Drawing from the well-established working memory task, the *N*-back [48], participants will determine if the current letter presented on the screen is the same or different from the letter displayed immediately before (*N* = 1, 1back). Participants will press the left arrow key to indicate the letters are different and the right arrow key to indicate the letters are the same. Sequences will be used in which the same response is correct for approximately 30% of stimuli.

For both tasks, the participant will be able to change from one response to another without penalty, though each key press is recorded. Responses after the stimulus is no longer displayed are recorded, and the final response prior to the display of the next stimulus is the one that is scored as correct or incorrect.

#### Questionnaire measures of cognitive effort in the cognitive effort tasks

##### Variant Questionnaire:

Includes 2 items rated using a 7-item Likert scale. Both will be asked after each of the six ISI-Stimulus Duration variants in the Variant Task: “How much did you like doing the task?” (1 = “very unpleasant”, 4 = “neutral” and 7 = “very enjoyable”) and “How much mental effort was required?” (1 = “none” and 7 = “a lot”).

##### Cognitive Effort Rating Scale:

Includes 4 items assessing perceived task liking (“very unpleasant” to “pleasant”), discomfort (“none” to “a lot”), cognitive effort required (“none” to ‘a lot”), and confidence in their performance with ≥80% accuracy (“absolutely sure I could not” to “absolutely sure I could”) rated using a 7-point Likert scale. Participants will answer these 4 questions while imagining they had performed the cognitive effort task for four durations (1, 5, 10 and 20 min).

#### Cognitive effort discounting task

The task is based on delay discounting tasks developed previously by Mitchell [2,3,49]. Participants are asked “At this moment, what would you prefer?” and choose between two alternatives: (1) a large amount ($10, $25 or $50) available for performing cognitive effort for some specific duration (1, 5, 10 or 20 min) and (2) a small amount of money available with no effort (12 values spanning $0 to a value larger than the effort-requiring amount: $11, $26, $55). This yields 144 questions (3 amounts, 4 effort levels, 12 no-effort values). For each question, we will record the preferred alternative, and the time taken to make the decision.

#### Eye tracking and pupillometry monitoring

Eye movements and pupil size will be collected during the Variant Task and the Cognitive Effort Discounting Task for each type of cognitive effort using a Gazepoint GP3 HD eye tracker (https://www.gazept.com/) running Gazepoint Control Panel v.6 and iMotions v8.2 (https://imotions.com/).

All periods of eye-tracking begin a 9-point iMotions-based calibration. Baseline pupil size data are recorded during a countdown period preceding each of the six ISI-Stimulus Duration variants of the Variant Task (<5000 ms) and during the blank screen (1000 ms) prior to each choice question during the Cognitive Effort Discounting Task. These baseline data will be used as a correction factor when calculating pupil sizes in response to the putatively different effort levels required in the Variant Task and during decision-making in the Cognitive Effort Discounting Task. We will also record eye movements and gaze durations during the Cognitive Effort Discounting Task to determine: (1) which alternative is inspected first, (2) number of times each is inspected, (3) the time spent gazing at each, and (4) time spent gazing at other locations on the screen.

## DATA ANALYSIS PLAN

### Sample Size and Power Analysis

With the assistance of Dr. Nigg’s study team, we will identify and recruit 96 healthy controls and 96 ADHD-diagnosed individuals. Each group will be 50% female so that analyses can include gender as a factor. Because these individuals have detailed demographic histories from the parent study, groups should be roughly similar in terms of psychopathological characteristics other than ADHD diagnosis.

Power analyses, driven by Aim 1 (determine whether ADHD is associated with heightened discounting of larger, more effortful rewards than healthy controls), were performed using preliminary data from the Cognitive Effort Discounting Task. GPower software indicated that a sample of 48 subjects/group would be sufficient to enable us to detect small-to-moderate effect sizes (*d* = 0.2, α = 0.05, 1 − β = 0.8) using between group ANOVA, coupled with post hoc tests to examine group and gender differences. Moderate effect sizes seem likely given that effect sizes in delay discounting studies are >0.4 [19]. Further, group sample sizes (*N* = 96) would allow the use of multiple mediator models to examine relations amongst variables in Aim 3, if gender were disregarded.

### Aim 1 Analysis Plan

Quantification of the Cognitive Effort Discounting Task will use procedures developed in our lab [2]: the amount of money at which participants are indifferent between effortless money (“reward with no effort”) and effortful money will be calculated (the indifference point) for each effort level (1, 5, 10 and 20 min). Indifference points will be examined for systematicity [50]. Individuals with nonsystematic functions may be removed, though ADHD may be associated with a larger incidence of nonsystematic data, which would deplete our sample sizes and cause us to simplify our analyses, perhaps by collapsing over gender. However, delay discounting data generated during the parent study [51,52] has shown similar levels of nonsystematicity between ADHD and healthy control samples, so this is not viewed as a concern. To better quantify the effort discounting function, we will fit a hyperbolic function [53], which is often used to describe delay discounting. At this time, it is unclear that individuals’ indifference points would be best described by this function or some other (e.g., Hartmann et al. [54] suggest parabolic functions are superior for physical effort discounting), so we will calculate the normalized area under the discounting curve [AUC], as described by Myerson et al. [55], and may explore other function fits as outlined in the literature [9,56].

Depending on goodness-of-fit indices (e.g., Akaike Information Criterion), a determination about the most descriptive index of discounting will be made. ANOVAs (between groups: Group and Gender) and within subjects (Reward size) will be used to examine whether ADHD is associated with heightened discounting of larger, more effortful rewards than healthy controls in these tasks that have been matched for subjective effort ratings, and whether these differences are moderated by Gender and/or by Reward size. To explore whether performance accuracy during the 1 minute practice contributes to effort discounting, ANCOVAs, with accuracy as the covariate, will also be conducted with the same factor structure as the primary ANOVAs. If appropriate discounting summary indices cannot be identified, mixed model logistic regression models will be used [57] to examine factors accounting for choice of the small, no-effort or the larger, effort requiring alternative (Group, Gender, Reward, Effort level). Cognitive effort types will be examined separately, though differences are not anticipated, similar findings for each will enhance the robustness of conclusions that can be drawn about factors influencing choice. Prediction: ADHD will be associated with heightened discounting over a range of rewards sizes compared to healthy controls, even when parameters are adjusted so both groups rate the effort required as equally subjectively demanding.

### Aim 2 Analysis Plan

Using an analysis pipeline similar to Hsu et al. [24,27], we will analyze the Likert data generated for perceived effort required and task liking, using mixed factor ANOVAs with Group (ADHD/healthy control), Gender (male/female), ISI and Stimulus Duration as factors, for each type of cognitive effort. These analyses will allow us to examine whether the subjective perception of effort in youth diagnosed with ADHD is higher than for healthy controls under the same ISI and Stimulus Duration conditions. Regression models will be used to examine the contributions of discomfort and effort exerted to task liking for each Group and Gender.

#### Prediction:

ADHD individuals will rate the subjective effort involved in these tasks as higher than controls at equivalent objective effort requirements. Analyses of these subjective ratings will indicate that there are Group × Gender × Effort Level interactions, rather than a simple Group-based effect.

### Aim 3 Analysis Plan

Summary indices of effort discounting will be used from the Aim 1 analysis. Group and gender differences will be assessed using ANOVAs on the trait measures of willingness to exert effort (grit, distress tolerance, etc.), pupil sizes and gaze durations will be calculated for periods when subjects are gazing at the effortful alternative and it was subsequently chosen or rejected. Supplementary ANOVAs will examine the effects of Effort level and Reward size on pupil size and gaze durations to determine whether group and gender moderate the effects of these variables as might be expected from literature [58]. If Groups differ considerably on subjective effort ratings for the cognitive effort task, a regression approach will be substituted that includes this rating as a covariate. To explore relations amongst indices of effort discounting, theoretically-related traits, pupillometry and gaze data, and measures of cognitive function assessed on a separate session as part of the Nigg research program, multiple mediator models will be created to identify factors contributing to effort discounting, and whether models are independent of Reward Size and Effort type. Depending on final sample characteristics, age may be included in these models. By creating a model for one effort type, we can assess its ability to fit data for the other cognitive effort type, enhancing the generality of our conclusions if the same factors influence behavior, and raising more questions and directions for future behavioral and nosological research if they do not. Prediction: trait variations will not account for differences in cognitive effort discounting, but relationships with other cognitive capacities and pupillometry data will identify factors associated with heightened effort aversion and altered Reward × Effort Level interactions for both ADHD and healthy control groups, and genders.

## DISCUSSION

The research proposed for this study includes three highly innovative elements to explore the dynamics of cognitive effort in pursuit of a better understanding of ADHD and possibly to other psychopathologies characterized by limitations of their willingness to exert cognitive effort.

First, while interest in effort discounting is growing, most existing tasks vary effort by making the task requirements more difficult in ways that simultaneously increase the possibility of failure [22]. This research project focuses on increasing the duration that an executive function must be engaged, innovatively manipulating effort while ensuring that selection of the high effort alternative is not driven by a fear of inaccurate performance resulting in not earning the larger reward. This method also has more relevance for the concerns raised in connection to ADHD-diagnosed individuals and their inability to remain on-task. The experimental approach in the proposed research, i.e., the examination of the role of willingness to exert cognitive effort on ADHD-diagnosed individuals and healthy controls, is highly novel. As noted earlier, to our knowledge, in ADHD, only one study has examined physical effort-related decision-making [8] but no studies have examined cognitive effort. Consequently, successfully completing Aim 1 (determine whether ADHD is associated with heightened discounting of larger, more effortful rewards than healthy controls) supports future research to explore the psychosocial, neural, and genetic mechanisms underlying the differences between willingness to exert effort in ADHD, but also in other psychopathologies, and case-matched, healthy controls.

Second, while the theoretical concept of cognitive [mental] effort has been previously discussed in connection with psychopathology and is recognized in the RDoC, very little research has attempted to move this concept from the qualitative to the quantitative realm, as proposed in this study. The methodology under-development in this project is highly innovative with the intention of elucidating the mechanisms underlying the concerns faced by these clinical populations in a novel way. By systematically examining the effect of changes in the ISI and Stimulus Duration parameters on performance on the cognitive effort tasks, as well as the subjective ratings of effort, we hope to provide a methodology to permit responses to sustained attention and working memory loads to be quantified under Aim 2 (examine the subjective perception of cognitive effort in youth diagnosed with ADHD and healthy controls). It is possible that participants will be insensitive to differences between the six combinations of ISI and Stimulus Durations, and will rate all as requiring similar levels of cognitive effort. To guard against this possibility, we plan to compare the subjective ratings of effort for the six variants after 18 participants have been completed using tests of equivalence [59]. If variants are rated similarly, we will shorten the shortest ISI by 50 ms and lengthen the longest Stimulus Duration by 50 ms, and reassess variant ratings after further 18 participants. This adjustment algorithm will be applied until ratings of subjective effort for the different variants are no longer similar.

Third, successful completion of Aim 3 (examine relationships amongst indices of effort discounting, theoretically-related traits, biomarkers of effort-related decision-making and measures of cognitive function from the parent study) will tie the results to the larger ADHD literature and provide additional scientific justification for future studies of neural mechanisms and interventions. Further, it is our hope that the inclusion of two distinct cognitive tasks (sustained attention and working memory) in conjunction with the range of cognitive measures from the parent study may provide insight into the largely entangled executive function processes underlying both tasks, providing a more nuanced view of the cognitive markers of ADHD and the cognitive limitations that vary widely across individuals with the disorder.

The study design has a number of potential limitations. First, as with any quasi-experimental study, the ADHD-diagnosed and healthy control individuals may differ in ways that are related to their group membership that affect cognitive effort discounting, for example, differences in the types and severity of co-morbid conditions like depression and co-occurring use of substances/medications. The Health Questionnaire will provide some data about these sources of group differences and permit some exploratory analyses of the robustness of effects when participants are omitted. However, such analyses would only provide a foundation to justify future research, rather than provide a definite answer to the role of these comorbidities in differences in effort discounting. A second study limitation is that if group differences in effort discounting are primarily due to differences in perceived difficulty, our plan to control for perceived difficulty level by matching groups on these ratings may eliminate group differences in cognitive effort discounting. However, such a lack of differences is still informative and suggest that the neuropsychological processes involved in devaluation of effortful rewards are not different between groups; rather differences reported outside the laboratory are driven by ADHD-diagnosed individuals perceiving the cognitive effort costs as higher.

## CONCLUSIONS

A “dislike of mental effort” is a diagnostic criteria for ADHD, a trait closely associated to attention and working memory (constructs in the RDoC domain: Cognitive Systems and Reward Valuation; subconstruct: Effort [1]). However other disorders are also characterized by related constructs such as cognitive fatigue and apathy, such as depression, making a disrupted willingness to exert cognitive effort a potentially trans-disease process. This project will provide a quantitative description of cognitive effort-related decision-making that can be used to advance our understanding of ADHD, including identifying distinctions between different ADHD subtypes, as well as these other disorders. We anticipate that this research will provide a foundation on which to create more refined methodologies to examine willingness to engage in cognitive effort that can easily be implemented in other psychiatric populations.

## Figures and Tables

**Table 1.** Study inclusion and exclusion criteria.

Inclusion criteria
Currently enrolled in “ADHD heterogeneity, mechanisms, and risk profile” research study
Classified as a healthy control OR meeting DSM-5 [30] diagnostic criteria for ADHD by Dr. Nigg’s study team during recruitment to that study; verified at most recent visit for that study
Aged 16–21 years
Willing to undergo 48-h washout period for all ADHD medications prior to study visit
At least 8th grade English speaking and reading ability
Exclusion criteria
Extensive nicotine use: smoke 10 or more cigarettes daily or vape nicotine daily
Currently pregnant
Substance use within 24 h of study visit: alcohol, amphetamine, methamphetamine, cocaine, benzodiazepines, or opiates

## ABBREVIATIONS

ADHD: Attention Deficit Hyperactivity Disorder
ISI: Inter-stimulus interval
ms: milliseconds
NIMH: National Institute of Mental Health
RDoC: Research Domain Criteria

## References

[1] National Institute of Mental Health. RDoC Constructs U.S.Department of Health and Human Services. Available from: https://www.nimh.nih.gov/research/research-funded-by-nimh/rdoc/constructs/index.shtml. Accessed 2020 Dec 23.

[2] Mitchell SH. Measures of impulsivity in cigarette smokers and non-smokers. Psychopharmacology. 1999;146(4):455–64. doi: 10.1007/pl00005491 10550496

[3] Mitchell SH. Effects of short-term nicotine deprivation on decision-making: delay, uncertainty and effort discounting. Nicotine Tob Res. 2004;6(5):819–28. doi: 10.1080/14622200412331296002 15700917

[4] Mitchell SH. Devaluation of Outcomes Due to Their Cost: Extending Discounting Models Beyond Delay In: Stevens JR, editor. Impulsivity. Nebraska Symposium on Motivation. Cham (Switzerland): Springer International Publishing; 2017 p. 145–61. 30351562

[5] Mitchell SH. Discounting the Value of Commodities According to Different Types of Cost In: Vuchinich RE, Heather N, editors. Choice, Behavioural Economics and Addiction. Amsterdam (The Netherlands): Pergamon; 2003 p. 339–62.

[6] Libedinsky C, Massar SA, Ling A, Chee W, Huettel SA, Chee MW. Sleep deprivation alters effort discounting but not delay discounting of monetary rewards. Sleep. 2013;36(6):899–904. doi: 10.5665/sleep.2720 23729933 PMC3649832

[7] Botvinick MM, Huffstetler S, McGuire JT. Effort discounting in human nucleus accumbens. Cogn Affect Behav Neurosci. 2009;9(1):16–27. doi: 10.3758/CABN.9.1.16 19246324 PMC2744387

[8] Mies GW, Ma I, de Water E, Buitelaar JK, Scheres A. Waiting and working for rewards: Attention-Deficit/Hyperactivity Disorder is associated with steeper delay discounting linked to amygdala activation, but not with steeper effort discounting. Cortex. 2018;106:164–73. doi: 10.1016/j.cortex.2018.05.018 30005368

[9] Bialaszek W, Marcowski P, Ostaszewski P. Physical and cognitive effort discounting across different reward magnitudes: Tests of discounting models. PLoS One. 2017;12(7):e0182353. doi: 10.1371/journal.pone.0182353 28759631 PMC5536267

[10] Luman M, Tripp G, Scheres A. Identifying the neurobiology of altered reinforcement sensitivity in ADHD: a review and research agenda. Neurosci Biobehav Rev. 2010;34(5):744–54. doi: 10.1016/j.neubiorev.2009.11.021 19944715

[11] Tenenbaum RB, Musser ED, Raiker JS, Coles EK, Gnagy EM, Pelham WE Jr. Specificity of Reward Sensitivity and Parasympathetic-Based Regulation among Children with Attention-Deficit/Hyperactivity and Disruptive Behavior Disorders. J Abnorm Child Psychol. 2018;46(5):965–77. doi: 10.1007/s10802-017-0343-0 28875352 PMC5839917

[12] Tucha L, Fuermaier AB, Koerts J, Buggenthin R, Aschenbrenner S, Weisbrod M, Sustained attention in adult ADHD: time-on-task effects of various measures of attention. J Neural Transm. 2017;124(Suppl 1):39–53. doi: 10.1007/s00702-015-1426-0 26206605 PMC5281679

[13] Salomone S, Fleming GR, Bramham J, OʼConnell RG, Robertson IH. Neuropsychological Deficits in Adult ADHD: Evidence for Differential Attentional Impairments, Deficient Executive Functions, and High Self-Reported Functional Impairments. J Atten Disord. 2020;24(10):1413–24. doi: 10.1177/1087054715623045 26769747

[14] Marchetta ND, Hurks PP, Krabbendam L, Jolles J. Interference control, working memory, concept shifting, and verbal fluency in adults with attention-deficit/hyperactivity disorder (ADHD). Neuropsychology. 2008;22(1):74–84. doi: 10.1037/0894-4105.22.1.74 18211157

[15] Cockcroft K Working memory functioning in children with attention-deficit/hyperactivity disorder (ADHD): A comparison between subtypes and normal controls. J Child Adolesc Ment Health. 2011;23(2):107–18. doi: 10.2989/17280583.2011.634545 25860085

[16] Nigg JT. Annual Research Review: On the relations among self-regulation, self-control, executive functioning, effortful control, cognitive control, impulsivity, risk-taking, and inhibition for developmental psychopathology. J Child Psychol Psychiatry. 2017;58(4):361–83. doi: 10.1111/jcpp.12675 28035675 PMC5367959

[17] Westbrook A, Lamichhane B, Braver T. The Subjective Value of Cognitive Effort is Encoded by a Domain-General Valuation Network. J Neurosci. 2019;39(20):3934–47. doi: 10.1523/JNEUROSCI.3071-18.2019 30850512 PMC6520500

[18] Madden GJ, Johnson PS. A delay-discounting primer In Madden GJ, Bickel WK, editors. Impulsivity: The behavioral and neurological science of discounting. Washington (DC, US): American Psychological Association; 2010 p. 11–37.

[19] Jackson JN, MacKillop J. Attention-Deficit/Hyperactivity Disorder and Monetary Delay Discounting: A Meta-Analysis of Case-Control Studies. Biol Psychiatry Cogn Neurosci Neuroimaging. 2016;1(4):316–25. doi: 10.1016/j.bpsc.2016.01.007 27722208 PMC5049699

[20] Marx I, Hacker T, Yu X, Cortese S, Sonuga-Barke E. ADHD and the Choice of Small Immediate Over Larger Delayed Rewards: A Comparative Meta-Analysis of Performance on Simple Choice-Delay and Temporal Discounting Paradigms. J Atten Disord. 2018:1087054718772138. doi: 10.1177/1087054718772138 29806533

[21] Cocker PJ, Hosking JG, Benoit J, Winstanley CA. Sensitivity to cognitive effort mediates psychostimulant effects on a novel rodent cost/benefit decision-making task. Neuropsychopharmacology. 2012;37(8):1825–37. doi: 10.1038/npp.2012.30 22453140 PMC3376315

[22] Westbrook A, Kester D, Braver TS. What is the subjective cost of cognitive effort? Load, trait, and aging effects revealed by economic preference. PLoS One. 2013;8(7):e68210. doi: 10.1371/journal.pone.0068210 23894295 PMC3718823

[23] Ostaszewski P, Bąbel P, Swebodziński B. Physical and cognitive effort discounting of hypothetical monetary rewards. Jpn Psychol Res. 2013;55(4):n/a–n/a. doi: 10.1111/jpr.12019

[24] Hsu CF, Eastwood JD, Toplak ME. Differences in Perceived Mental Effort Required and Discomfort during a Working Memory Task between Individuals At-risk And Not At-risk for ADHD. Front Psychol. 2017;8:407. doi: 10.3389/fpsyg.2017.00407 28377736 PMC5359313

[25] Gronwall DM. Paced auditory serial-addition task: a measure of recovery from concussion. Percept Mot Skills. 1977;44(2):367–73. doi: 10.2466/pms.1977.44.2.367 866038

[26] Kessler RC, Adler L, Ames M, Demler O, Faraone S, Hiripi E, The World Health Organization Adult ADHD Self-Report Scale (ASRS): a short screening scale for use in the general population. Psychol Med. 2005;35(2):245–56. doi: 10.1017/s0033291704002892 15841682

[27] Hsu CF, Propp L, Panetta L, Martin S, Dentakos S, Toplak ME, Mental effort and discomfort: Testing the peak-end effect during a cognitively demanding task. PLoS One. 2018;13(2):e0191479. doi: 10.1371/journal.pone.0191479 29432429 PMC5809041

[28] Duckworth AL, Peterson C, Matthews MD, Kelly DR. Grit: perseverance and passion for long-term goals. J Pers Soc Psychol. 2007;92(6):1087–101. doi: 10.1037/0022-3514.92.6.1087 17547490

[29] Simons JS, Gaher RM. The Distress Tolerance Scale: Development and Validation of a Self-Report Measure. Motiv Emot. 2005;29(2):83–102. doi: 10.1007/s11031-005-7955-3

[30] American Psychiatric Association. Diagnostic and statistical manual of mental disorders: DSM-5. 5th ed. Arlington (VA, US): American Psychiatric Association; 2013.

[31] MacKillop J, Amlung MT, Few LR, Ray LA, Sweet LH, Munafo MR. Delayed reward discounting and addictive behavior: A meta-analysis. Psychopharmacology. 2011;216(3):305–21. doi: 10.1007/s00213-011-2229-0 21373791 PMC3201846

[32] Amlung M, Vedelago L, Acker J, Balodis I, MacKillop J. Steep delay discounting and addictive behavior: A meta-analysis of continuous associations. Addiction. 2017;112(1):51–62. doi: 10.1111/add.13535 PMC5148639 27450931

[33] Brandstätter E, Brandstätter H. What’s money worth? Determinants of the subjective value of money. J Econ Psychol. 1996;17(4):443–64. doi: 10.1016/0167-4870(96)00019-0

[34] Horne JA, Ostberg O. A self assessment questionnaire to determine morningness eveningness in human circadian rhythms. Int J Chronobiol. 1976;4:97–110. 1027738

[35] Adler NE, Epel ES, Castellazzo G, Ickovics JR. Relationship of subjective and objective social status with psychological and physiological functioning: preliminary data in healthy white women. Health Psychol. 2000;19(6):586–92. doi: 10.1037//0278-6133.19.6.586 11129362

[36] Serpell L, Waller G, Fearon P, Meyer C. The roles of persistence and perseveration in psychopathology. Behav Ther. 2009;40(3):260–71. doi: 10.1016/j.beth.2008.07.001 19647527

[37] Cacioppo JT, Petty RE. The need for cognition. J Pers Soc Psychol. 1982;42(1):116–31. doi: 10.1037/0022-3514.42.1.116

[38] Cacioppo JT, Petty RE, Kao CF. The efficient assessment of need for cognition. J Pers Assess. 1984;48(3):306–7. doi: 10.1207/s15327752jpa4803_13 16367530

[39] Nigg JT. The ADHD response-inhibition deficit as measured by the stop task: replication with DSM-IV combined type, extension, and qualification. J Abnorm Child Psychol. 1999;27(5):393–402. doi: 10.1023/a:1021980002473 10582840

[40] Schachar R, Tannock R, Marriott M, Logan G. Deficient inhibitory control in attention deficit hyperactivity disorder. J Abnorm Child Psychol. 1995;23(4):411–37. doi: 10.1007/BF01447206 7560554

[41] De Luca CR, Wood SJ, Anderson V, Buchanan JA, Proffitt TM, Mahony K, Normative data from the CANTAB. I: development of executive function over the lifespan. J Clin Exp Neuropsychol. 2003;25(2):242–54. doi: 10.1076/jcen.25.2.242.13639 12754681

[42] Curko Kera EA, Marks DJ, Berwid OG, Santra A, Halperin JM. Self-report and objective measures of ADHD-related behaviors in parents of preschool children at risk for ADHD. CNS Spectr. 2004;9(9):639–47. doi: 10.1017/s1092852900001917 15337861

[43] Cornblatt BA, Malhotra AK. Impaired attention as an endophenotype for molecular genetic studies of schizophrenia. Am J Med Genet. 2001;105(1):11–5. 11424979

[44] Wing AM, Kristofferson AB. The timing of interresponse intervals. Percept Psychophys. 1973;13(3):455–60. doi: 10.3758/bf03205802

[45] Wing AM, Kristofferson AB. Response delays and the timing of discrete motor responses. Percept Psychophys. 1973;14(1):5–12. doi: 10.3758/bf03198607

[46] Wing AM. Voluntary timing and brain function: an information processing approach. Brain Cogn. 2002;48(1):7–30. doi: 10.1006/brcg.2001.1301 11812030

[47] Wechsler D Wechsler Intelligence Scale for Children. 4th ed. San Antonio (TX, US): Psychological Corporation; 2003.

[48] Kirchner WK. Age differences in short-term retention of rapidly changing information. J Exp Psychol. 1958;55(4):352–8. doi: 10.1037/h0043688 13539317

[49] Mitchell SH, Wilson VB. Differences in delay discounting between smokers and nonsmokers remain when both rewards are delayed. Psychopharmacology (Berl). 2012;219(2):549–62. doi: 10.1007/s00213-011-2521-z 21983917 PMC3677053

[50] Johnson MW, Bickel WK. An algorithm for identifying nonsystematic delay-discounting data. Exp Clin Psychopharmacol. 2008;16(3):264–74. doi: 10.1037/1064-1297.16.3.264 18540786 PMC2765051

[51] Karalunas SL, Gustafsson HC, Dieckmann NF, Tipsord J, Mitchell SH, Nigg JT. Heterogeneity in development of aspects of working memory predicts longitudinal attention deficit hyperactivity disorder symptom change. J Abnorm Psychol. 2017;126(6):774–92. doi: 10.1037/abn0000292 28782975 PMC5657320

[52] Wilson VB, Mitchell SH, Musser ED, Schmitt CF, Nigg JT. Delay discounting of reward in ADHD: application in young children. J Child Psychol Psychiatry. 2011;52(3):256–64. doi: 10.1111/j.1469-7610.2010.02347.x 21083561 PMC3059765

[53] Mazur JE. An adjusting procedure for studying delayed reinforcement In: Commons ML, Mazur JE, Nevin JA, Rachlin H, editors. Quantitative Analyses of Behavior. 5. Hillsdale (NJ, US): Earlbaum; 1987 p. 55–73.

[54] Hartmann MN, Hager OM, Tobler PN, Kaiser S. Parabolic discounting of monetary rewards by physical effort. Behav Processes. 2013;100:192–6. doi: 10.1016/j.beproc.2013.09.014 24140077

[55] Myerson J, Green L, Warusawitharana M. Area under the curve as a measure of discounting. J Exp Anal Behav. 2001;76(2):235–43. doi: 10.1901/jeab.2001.76-235 11599641 PMC1284836

[56] Mitchell SH, Wilson VB, Karalunas SL. Comparing hyperbolic, delay-amount sensitivity and present-bias models of delay discounting. Behav Processes. 2015;114:52–62. doi: 10.1016/j.beproc.2015.03.006 25796454 PMC4404224

[57] Kirkpatrick K, Marshall AT, Steele CC, Peterson JR. Resurrecting the individual in behavioral analysis: Using mixed effects models to address nonsystematic discounting data. Behav Anal. 2018;18(3):219–38. doi: 10.1037/bar0000103 PMC6101656 30135865

[58] van der Wel P, van Steenbergen H. Pupil dilation as an index of effort in cognitive control tasks: A review. Psychon Bull Rev. 2018;25(6):2005–15. doi: 10.3758/s13423-018-1432-y 29435963 PMC6267528

[59] Lakens D, Scheel AM, Isager PM. Equivalence Testing for Psychological Research: A Tutorial. Adv Methods Pract Psychol Sci. 2018;1(2):259–69. doi: 10.1177/2515245918770963

